# The First Line of Defense: Receptor-like Protein Kinase-Mediated Stomatal Immunity

**DOI:** 10.3390/ijms23010343

**Published:** 2021-12-29

**Authors:** Zhe Wang, Xiaoping Gou

**Affiliations:** Ministry of Education Key Laboratory of Cell Activities and Stress Adaptations, School of Life Sciences, Lanzhou University, Lanzhou 730000, China; wangzh13@lzu.edu.cn

**Keywords:** ABA, RLKs, ROS, signal transduction, stomatal immunity

## Abstract

Stomata regulate gas and water exchange between the plant and external atmosphere, which are vital for photosynthesis and transpiration. Stomata are also the natural entrance for pathogens invading into the apoplast. Therefore, stomata play an important role in plants against pathogens. The pattern recognition receptors (PRRs) locate in guard cells to perceive pathogen/microbe-associated molecular patterns (PAMPs) and trigger a series of plant innate immune responses, including rapid closure of stomata to limit bacterial invasion, which is termed stomatal immunity. Many PRRs involved in stomatal immunity are plasma membrane-located receptor-like protein kinases (RLKs). This review focuses on the current research progress of RLK-mediated signaling pathways involved in stomatal immunity, and discusses questions that need to be addressed in future research.

## 1. Introduction

Guard cells are a kind of specialized kidney-shaped epidermal cells. The pore between a pair of guard cells is called a stoma, meaning mouth in Greek. Stomata exist in the epidermis of leaves, stems, petals, sepals, and other organs of most terrestrial plants. The term “stomata” in botany generally refers to the stomatal complex including two guard cells and the pore between them [1,2]. Stomata are channels for plants to exchange gas and water with the external atmosphere. Therefore, stomata play a key role during photosynthesis and transpiration in plants by regulating stomatal aperture to govern gas exchange ratio or water loss. Many environmental stimuli can induce stomatal movement, such as relative humidity [3], drought [4], CO_2_ concentration [5,6], and light [3]. Stomatal aperture is also regulated by plant hormones. For example, abscisic acid (ABA) regulates stomatal movement under drought conditions or bacteria invasion by inducing the production of reactive oxygen species (ROS) and a transient increase of cytosolic Ca^2+^ ([Ca^2+^]_cyt_) in guard cells [7,8]. Salicylic acid (SA) regulates stomatal aperture by mediating ROS production in Arabidopsis (*Arabidopsis thaliana*) guard cells [9,10]. Methyl jasmonate (MeJA) can activate Ca^2+^ permeable cation channels in guard cells to close the stomata, which is similar to the mechanism of ABA-regulated stomatal closure [11,12].

**Figure 1:**
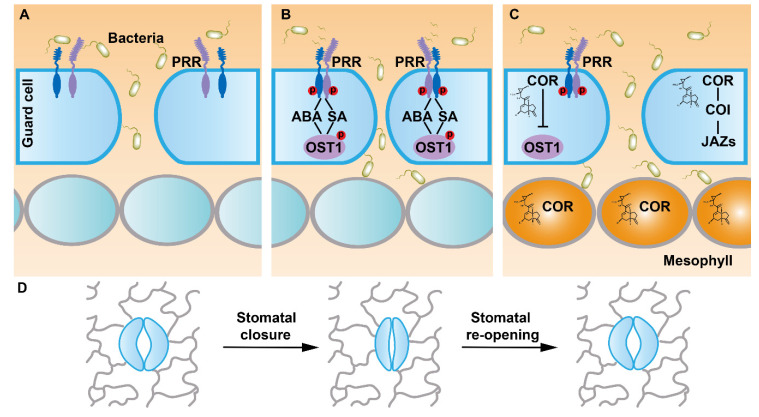
A general model of stomatal movement in plant defense against bacteria. The stomata are formed by two guard cells and the pore between them. The stomata are the main entry point of pathogen invasion. (**A**) When bacteria such as *Pseudomonas syringae* pv. *tomato* DC3000 (*Pst* DC3000) invade plants, pathogen-associated molecule patterns (PAMPs) can be sensed by membrane-localized pattern recognition receptors (PRRs) such as FLAGELLIN-SENSING 2 (FLS2). (**B**) FLS2 recruits its co-receptor BRI1-ASSOCIATED RECEPTOR KINASE 1 (BAK1) after perception of the PAMP to trigger rapid stomatal closure and limit bacterial invasion through ABA/SA signaling and a downstream kinase OPEN STOMATA 1 (OST1). (**C**) *Pst* DC3000 releases phytotoxin coronatine (COR) which is a structural mimic of JA conjugated to isoleucine (JA-Ile) to re-open stomata through the JA signaling pathway for more bacteria to infect plants. (**D**) A schematic diagram of stomatal movement during bacteria invasion.

When bacteria infect plants, they can survive on the plant surface and attach to stomata via their pili [13,14]. Numerous stomata are widely distributed on the epidermis, which therefore are the major way for the entry of bacteria. In the past two decades, researchers revealed that stomata are involved in plant innate immunity [15]. *Pseudomonas syringae* pv. *tomato* DC3000 (hereafter *Pst* DC3000) is a virulent pathogen of Arabidopsis and tomato (*Solanum lycopersicum*). Treatment with *Pst* DC3000 induces Arabidopsis stomatal closure, which can be reverted to the open state after the leaves are continuously treated with *Pst* DC3000 [15]. *E. coli* can trigger stomatal closure as well. However, continuous treatment with *E. coli* cannot re-open the stomata [15]. Therefore, although both plants and human bacteria can induce stomatal closure, *Pst* DC3000 acquired a specific mechanism during evolution to re-open stomata in order to facilitate more bacteria invading into the host plant. The phytotoxin coronatine (COR) is a well-characterized virulence factor of *Pst* DC3000. Lack of coronatine production reduces the toxicity of the mutant *Pst* bacteria [16], and the COR-deficient *Pst* DC3000 *cor^−^* fails to re-open the closed stomata [15]. COR is considered as a structural mimic of JA conjugated to isoleucine (JA-Ile), and COR exhibits biological functions similar to JA-Ile [17]. COR is able to bind to the same receptor of JA to re-open the closed stomata induced by bacteria attack through the JA signaling pathway [12,18] (Figure 1).

The plant under bacterial infection can sense the bacterial molecules such as flg22 (a synthetic peptide of flagellin) and lipopolysaccharide (LPS), and close stomata to limit bacteria entry. These bacterial molecules were defined as pathogen/microbe-associated molecular patterns (PAMPs) [19,20,21,22]. PAMPs are recognized by the pattern recognition receptors (PRRs) that are plasma membrane-localized proteins with varied structures and functions. Most PRRs are plasma membrane-localized receptor-like protein kinases (RLKs) [21,23]. RLKs transduce extracellular signals into the cell to coordinate cellular activities during plant development and response to environmental stimuli [24,25,26]. In the past two decades, the functions of RLKs in plant immunity were extensively studied. For instance, FLAGELLIN-SENSING 2 (FLS2), a leucine-rich repeat (LRR) RLK, and its co-receptor BRI1-ASSOCIATED RECEPTOR KINASE 1 (BAK1) trigger plant innate immunity upon the perception of flg22 [27,28,29]. FERONIA (FER), a malectin-like receptor kinase, positively regulates plant immunity by destabilizing MYC2, a core transcription factor in the JA signaling pathway [30]. The LysM receptor kinase CHITIN ELICITOR RECEPTOR KINASE 1/LYSM DOMAIN RECEPTOR-LIKE KINASE 1 (CERK1/LYK1) and other LYKs form a PRR complex to sense chitin and regulate plant immunity against fungal infection [31,32,33,34]. Arabidopsis L-type lectin receptor-like protein kinase (LecRK) family contains 45 members, many of which were reported to regulate plant innate immunity by sensing various signals, such as extracellular purine molecules and PAMPs [35,36,37]. It is worth noting that lines of evidence suggested that these RLKs mediate signals to regulate stomatal movement during plant immunity. In this review, we discuss recent findings on stomatal immunity, mainly focusing on RLK-mediated signaling and the downstream regulators involved in this process.

## 2. FLS2 Perceives Flg22 to Mediate Stomatal Immunity

Different from animals, plants cannot move away from adverse environments as sessile organisms, which makes plants more vulnerable to microbial pathogens. Terrestrial plants have developed two interactive immune systems in response to pathogenic bacteria during the long-term evolution process: PAMP-triggered immunity (PTI) and effector-triggered immunity (ETI) [22]. Plant PTI responses usually depend on phosphorylation cascades to trigger downstream cellular events. For example, flg22 can trigger defense responses in Arabidopsis, including production of ROS, activation of mitogen-activated protein kinases (MAPKs), increased [Ca^2+^]_cyt_, and induced expression of immunity-related genes [15,38,39,40,41]. It has been well recognized that FLS2 functions as the receptor of flg22 to trigger plant PTI and downstream immune responses [42,43].

**Figure 2:**
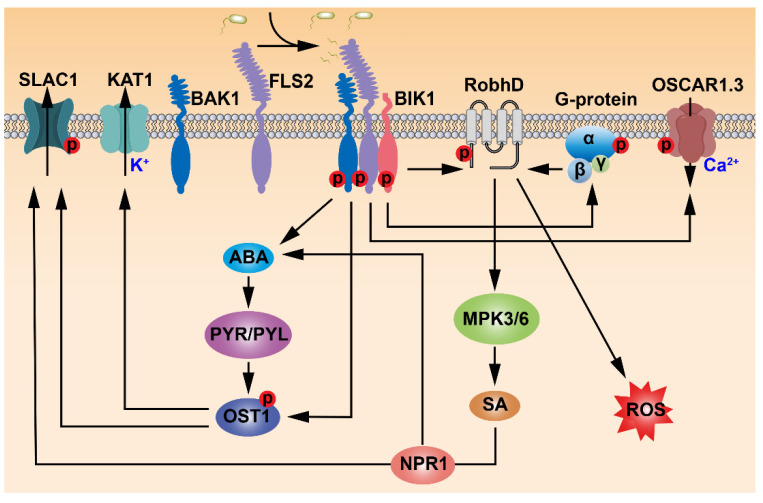
FLS2-mediated stomatal immunity. The FLS2 PRR complex plays a central role in *Pst* DC3000-triggered stomatal closure. FLS2 interacts with BAK1 and a receptor-like cytoplasmic kinase (RLCK) BOTRYTIS-INDUCED KINASE 1 (BIK1) to form a PRR complex in regulating stomatal immunity. BAK1 interacts with and phosphorylates OST1 to regulate ABA-induced stomatal closure by activating an S-type anion channel SLOW ANION CHANNEL-ASSOCIATED 1 (SLAC1) and a K^+^ channel in stomatal immunity. BIK1 interacts with and phosphorylates EXTRA-LARGE G PROTEINS (XLGs) to regulate RESPIRATORY BURST OXIDASE HOMOLOGUE D (RbohD)-mediated ROS production. BIK1 can also phosphorylate RbohD directly to control ROS production in stomatal immunity. The FLS2 PRR complex regulates stomatal closure through phosphorylating Ca^2+^-permeable channel OSCA1.3 by BIK1, increasing the channel activity of OSCA1.3. Salicylic acid (SA) accumulation is triggered by PRR and activates SLAC1 through NPR1-mediated signaling. The SA signaling can regulate ABA biosynthesis and positively modulate stomatal immunity.

**Figure 3:**
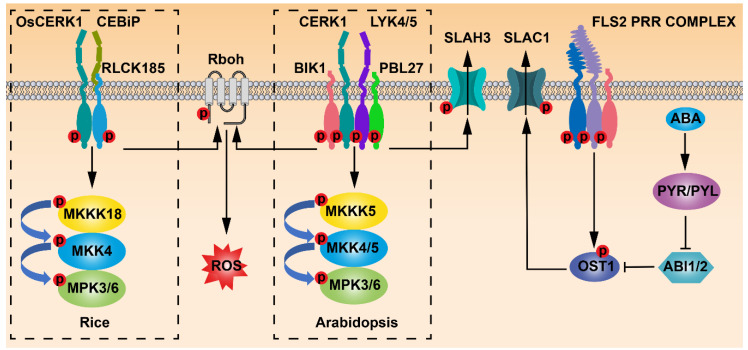
PRRs regulate stomatal immunity through different signaling pathways. Environmental factors regulate stomatal movement through ABA signaling pathway-mediated phosphorylation of OST1 and SLAC1. The flg22–FLS2 signaling phosphorylates OST1 to activate SLAC1 in stomatal immunity. The flg22 and ABA signaling pathways converge on OST1. CHITIN ELICITOR RECEPTOR KINASE 1 (CERK1) and its homologs LYSM DOMAIN RECEPTOR-LIKE KINASE 4/5 (LYK4/5) form a PRR complex to perceive the chitin signal to regulate stomatal immunity through the MKKK5–MKK4/5–MPK3/6 cascade in Arabidopsis. In rice, OsCERK1 and CHITIN OLIGOSACCHARIDE ELICITOR-BINDING PROTEIN (OsCEBiP) perceive the chitin signal to activate the MKKK18–MKK4–MPK3/6 cascade in stomatal immunity. In Arabidopsis, the RLCK PBS1-Like 27 (PBL27) can directly interact with and phosphorylate SLAC1 after it perceives the chitin–CERK1 signal to close stomata. BIK1 and OsRLCK185 directly phosphorylate Rboh to regulate ROS production during stomatal immunity in Arabidopsis and rice, respectively.

Perception of flg22 by FLS2 in Arabidopsis guard cells is necessary for bacterium-triggered stomatal closure. The stomata could not be closed in the *fls2* mutants treated with flg22 [15]. Moreover, the *fls2* mutants were able to rescue the virulence defects of COR-deficient *Pst* DC3118. *fls2* exhibited more susceptibility to *Pst* DC3118 than the wild type since they failed to close stomata in response to the infection. When *Pst* DC3118 was infiltrated directly into the leaf apoplast, *fls*2 and the wild-type plants showed similar susceptibility [44]. Thus, FLS2 plays a critical role in *Pst* DC3000-triggered stomatal closure. Flg22-induced and FLS2-mediated stomatal movement is mechanistically linked to SA and ABA signaling, including the guard cell-specific kinase OPEN STOMATA 1/SUCROSE NONFERMENTING 1-RELATED PROTEIN KINASE 2.6 (OST1/SnRK2.6) (Figure 1). In SA-deficient *nahG* transgenic plants and SA-biosynthetic mutant *enhanced disease susceptibility 16*/*salicylic acid induction deficient 2* (*eds16/sid2*), *Pst* DC3000 was not able to trigger stomatal closure. Similarly, flg22 could not induce stomatal closure in ABA signaling mutant *ost1-2* or ABA-deficient mutant *aba deficient 3* (*aba3-1*) [15]. Although exogenously applied ABA could induce stomatal closure responses in SA-deficient mutants, stomatal closure responses in ABA biosynthetic mutant *aba2-1* were insensitive to exogenous SA. On the other hand, SA treatment could not induce stomatal closure in *nonexpresser of pr genes 1-1* (*npr1-1*), an SA receptor mutant. Conversely, stomatal closure responses in *npr1-1* and *fls2* mutants were sensitive to exogenously applied ABA [44]. Taken together, SA acts upstream of ABA in flg22–FLS2-mediated stomatal closure. Flg22-triggered rapid stomatal closure was altered in *fls2* and *ost1*. In contrast, *aba insensitive 1-1* (*abi1-1*) was still sensitive to flg22-mediated rapid stomatal closure. Although both flg22 and ABA signaling activate the same anion channels through OST1 in regulating stomatal closure, it seems that the flg22–FLS2 and ABA signaling pathways diverge upstream of OST1 [45] (Figure 2 and Figure 3).

Many receptor RLKs require a different RLK functioning as a co-receptor to transduce varied extracellular signals [24]. BAK1 was the first identified co-receptor RLK, which functions together with the major brassinosteroid (BR) receptor BRASSINOSTEROID INSENSITIVE 1 (BRI1) to mediate BR signaling in regulating plant growth and development [46,47]. The water loss rate of *bak1* was higher than the wild type, and ABA could not induce stomatal closure in *bak1*, suggesting that BAK1 is required for ABA-triggered stomatal closure [48]. Biochemical assays revealed that BAK1 interacts with and phosphorylates the guard-cell-specific OST1 kinase to regulate ABA-induced stomatal closure. ABA could induce increased ROS levels in guard cells of the wild type, whereas ROS production in the *bak1* mutant was insensitive to exogenous ABA. These results indicated that BAK1 functions upstream of ROS production in ABA-induced stomatal closure [48]. The binding of flg22 to FLS2 induced the heteromerization and reciprocal phosphorylation between FLS2 and BAK1 during immune responses [27]. Moreover, BAK1 functions as a co-receptor of FLS2 in flg22-triggered immune response [27,28,29,49]. Taken together, these results suggest that FLS2 and BAK1 may form a receptor complex to regulate stomatal immunity (Figure 2).

Receptor-like cytoplasmic kinases (RLCKs) are a class of RLK that are anchored to the plasma membrane but lack the extracellular ligand-binding domain. RLCKs play essential roles in plant innate immunity, response to stresses, and development [50]. The Arabidopsis genome encodes a total of 149 RLCKs that were divided into 17 subfamilies based on their phylogeny [51]. The Arabidopsis RLCK VII subfamily consists of 46 members, and most of them are involved in PAMP-triggered immune signaling [52]. For example, BOTRYTIS-INDUCED KINASE 1 (BIK1) interacted with FLS2 to mediate PTI [53]. Flg22 treatment could induce calcium influx in the wild type, which depends on FLS2 [54,55]. However, the calcium influx level in *bik1* was only about half of that in the wild type when *bik1* was treated with flg22, which implied that BIK1 functions downstream of FLS2-mediated flg22 signaling [53]. ROS in plant guard cells plays a critical role during responses to pathogen attack or abiotic stress [10,56]. FLS2-mediated PTI is usually accompanied by a transient ROS burst, which depends on phosphorylation of RESPIRATORY BURST OXIDASE HOMOLOGUE D (RbohD), a nicotinamide adenine dinucleotide phosphate (NADPH) oxidase that is a key enzyme in generating ROS [53,57,58,59]. ROS production mediated by RbohD was necessary for flg22–FLS2-triggered stomatal movement during immunity to *Pseudomonas syringae* [53,60]. Biochemical results showed that RbohD directly interacted with BIK1 and FLS2, and BIK1 could phosphorylate RbohD at multiple sites. The phosphorylation of Ser39 and Ser343 in RbohD enhanced ROS production and was necessary for flg22-induced stomatal closure [53,60]. Calcium-dependent protein kinases (CPKs) were indicated to regulate ROS production during plant immunity [54,58,61]. Overexpression of *CPK5* could enhance the phosphorylation of Ser39 in RbohD during PAMP-mediated stomatal immunity [62]. However, LaCl_3_, a calcium channel blocker, could not inhibit the phosphorylation of Ser39 in RbohD, suggesting that phosphorylation of RbohD by BIK1 is independent of CPK5 [53]. Altogether, these data indicated that FLS2, BAK1, and BIK1 function together to regulate stomatal immunity through RbohD-mediated ROS production (Figure 2).

Heterotrimeric G proteins play a central role in signal transduction pathways of animals, which respond to various extracellular stimuli perceived by G protein-coupled receptors (GPCRs) [63]. Although no canonical GPCR has been identified in plants, similar heterotrimeric G proteins containing three subunits exist in plants. G PROTEIN ALPHA SUBUNIT 1 (GPA1), the Arabidopsis Gα was reported to be involved in ABA-mediated stomatal movement. The water loss rate of *gpa1* mutants was significantly higher than that of the wild-type plants because ABA failed to induce stomatal closure in *gpa1*. GPA1 was necessary for the inhibition of K^+^ influx into guard cells by ABA and sphingosine-1-phosphate (S1P), and the activation of anion channels by pH-independent ABA [64,65]. The flg22–FLS2 signaling could promote stomatal closure via inhibiting the K^+^ influx channels in guard cells. However, stomatal movement in *gpa1* was insensitive to flg22 treatment [66], implying that the flg22–FLS2 signaling regulates stomatal immunity through a GPA1-dependent pathway. In addition, plants encode specific extra-large G proteins (XLGs) which exhibit significant homology to animal and plant Gα subunits [67]. XLG2 directly interacted with the FLS2–BIK1 PRR complex in the pre-activation state, inhibiting the proteasome-mediated degradation of BIK1, together with the Gβ subunit AGB1 and Gγ subunits AGG1/2. When FLS2 perceived flg22, BIK1 phosphorylated the N terminus of XLG2 that then dissociated from the heterotrimeric G protein complex. The phosphorylated XLG2 could activate RbohD to produce ROS that induced stomatal closure [68]. These data demonstrated that G proteins are essential for FLS2-mediated stomatal movement in response to biotic stresses (Figure 2).

In terrestrial plants, the stomatal aperture is controlled by turgor changes caused by the transmembrane channel-regulated ion flux. For instance, the current change resulted by the transmembrane K^+^ flux regulates ABA- and flg22-induced stomatal closure [64,65]. Flg22 triggered membrane depolarization, which resulted in K^+^ efflux of guard cells and thus stomatal closure. In the *fls2* mutant, flg22 treatment did not change the K^+^ current of guard cells [66], suggesting that the flg22–FLS2 signaling regulates stomatal immunity through a K^+^ channel-dependent pathway. SLOW ANION CHANNEL-ASSOCIATED 1 (SLAC1) is an anion channel required for regulating stomatal responses to environment and pathogen stimuli. The S-type anion channels can transmit Cl^−^ and malate efflux in guard cells that cause stomatal closure by decreasing guard cell osmotic [69,70]. [Ca^2+^]_cyt_ and ABA could not activate stomatal closure in the *slac1* mutants, suggesting that SLAC1 mediates stomatal movement downstream of ABA and [Ca^2+^]_cyt_ [71,72]. SLAC1 also plays a role in PAMP-mediated stomatal immunity. A very low concentration of flg22 could activate SLAC1, whereas stomatal closure of the *slac1* mutant was less sensitive to flg22 treatment [45]. Recently, a Ca^2+^-permeable channel OSCA1.3 was identified in Arabidopsis, which was involved in FLS2-mediated stomatal immunity. OSCA1.3 could be activated rapidly with the treatment of flg22. BIK1 could interact with and phosphorylate the N-terminal of OSCA1.3, which activated the N-terminal and increased the channel activity of OSCA1.3 [73]. Taken together, the current knowledge supports that FLS2-mediated flg22 signaling requires transmembrane ion flux to regulate stomatal immunity (Figure 2).

## 3. CERK1-Mediated Chitin Signaling Regulates Stomatal Immunity

RLKs with lysin-motif (LysM) ectodomains were supposed to recognize specific molecules with *N*-acetylglucosamine, such as chitin, peptidoglycan (PGN), and rhizobial nodulation factor (NF). Chitin is a β-l, 4-linked homopolymer of *N*-acetylglucosamine, which mainly exists in the cell walls of most of the higher fungi [38]. Chitin is conferred as a PAMP that elicits plant immunity. Similar to flg22, treatment with chitin elicitor leads to stomatal closure [74], suggesting that stomatal immunity can be triggered by chitin. Mutation in CHITIN OLIGOSACCHARIDE ELICITOR-BINDING PROTEIN (OsCEBiP), the first plant chitin receptor identified in rice (*Oryza sativa*), resulted in the suppression of the elicitor-induced oxidative burst as well as the downstream gene responses [75]. OsCEBiP is a LysM-type receptor-like protein (LysM-RLP), which lacks a cytoplasmic kinase domain, implying that it must cooperate with another protein kinase to initiate chitin signaling. OsCERK1 was later identified to form a hetero-oligomeric receptor complex with OsCEBiP for perceiving chitin [76]. CERK1/LYK1 and LYK4/5 are the orthologs of OsCERK1 in Arabidopsis. CERK1 functions as chitin receptor to mediate chitin-triggered immunity [77,78]. The *lyk4 lyk5-2* double mutants exhibited complete insensitivity to chitin treatment, suggesting that LYK4/5 may form a complex with CERK1 to recognize chitin. In fact, chitin could induce LYK5 to interact with CERK1, and LYK5 binding to chitin was necessary for CERK1 phosphorylation [31]. Although chitoheptaose or chitin treatment could not induce the interaction between CERK1 and LYK4, the ectodomains of LYK4 and LYK5 showed physical interaction and LYK4 could form homodimers in vitro. Therefore, LYK4 may function as a co-receptor or scaffold protein of CERK1 to form the receptor complex of chitin in Arabidopsis [79,80].

RLCK and MAPK cascade play critical roles in chitin-triggered immunity. Silencing the expression of *OsRLCK185* interfered with chitin-mediated immunity responses, such as MAPK activation and downstream gene expression. OsRLCK185 interacted with and was phosphorylated by OsCERK1 [81]. OsRLCK185 was required for chitin-induced phosphorylation of OsMPK3 and OsMPK6 [81], suggesting that OsRLCK185 bridges the CERK1–CEBiP complex and MAPK cascade in chitin-triggered rice immunity. Moreover, chitin treatment enhanced the phosphorylation level of OsMPK6 by OsMKK4, and the kinase activities of OsMPK3 and OsMPK6 were activated by induced expression of constitutively activated OsMKK4 [82]. OsMKKK11 and OsMKKK18 were identified to interact with OsRLCK185 in vitro. Transient expression of *OsMKKK11* or *OsMKKK18* in *Nicotiana benthamiana* led to immune responses such as cell death. In addition, OsMKKK18 could be phosphorylated by OsRLCK185, and interaction between OsMKKK18 and OsMKK4 was detected with a yeast two-hybrid assay [83]. These data indicated that the OsMKKK18–OsMKK4–OsMPK3/6 signaling cascade transduces the chitin elicitor signal perceived by CERK1–CEBiP–OsRLCK185 to mediate rice immune responses.

A counterpart of chitin-triggered signaling cascade downstream of the receptor complex was also identified in Arabidopsis. PBS1-Like 27 (PBL27), an Arabidopsis ortholog of OsRLCK185, interacts with CERK1 at the plasma membrane. The *pbl27* mutants were less sensitive to chitin treatment, and the chitin-triggered activation of MPK3/6 was not observed in *pbl*27, which supported that a MAPK cascade containing MPK3/6 functions downstream of PBL27 to regulate chitin-mediated immunity in Arabidopsis [84]. An Arabidopsis MEKK subfamily member MKKK5 was identified to interact with PBL27 both in vivo and in vitro. The in vitro kinase assay revealed that PBL27 phosphorylated the C-terminal domain of MKKK5. MKKK5 could interact with and phosphorylate MKK4 and MKK5. Consistently, the absence of MKKK5 significantly reduced MPK3/6 activation triggered by a chitin elicitor [85]. It was reported that the inducible double mutants of *mpk3/6* and *mkk4/5* were more susceptible to pathogens than the wild type because the stomata of the mutants failed to be closed during pathogen invasion [86], which suggested that MKK4/5 and MPK3/6 are required for PAMP-induced stomatal immunity. Taken together, these data implied that chitin may regulate stomatal immunity through the CERK1–PBL27–MKKK5–MKK4/5–MPK3/6 phospho-signaling pathway (Figure 3).

Recently, a study revealed that PBL27 directly binds with the anion channel SLAC1 homolog 3 (SLAH3) to regulate chitin-induced stomatal immunity [87]. SLAH3 interacted with PBL27 in vitro, and PBL27 could phosphorylate SLAH3 at S127 and S189. The mutants with S127A and S189A exhibited defective stomatal closure, which was insensitive to chitin treatment. Enhanced phosphorylation of SLAH3 by PBL27 was observed after the plants were treated with chitin, suggesting that the activation of SLAH3 by PBL27 depends on chitin stimulation. Consistently, S-type anion currents could be detected only when SLAH3 was co-injected with PBL27. The activation of SLAH3 by PBL27 was not suppressed by ABI1, whereas ABI1 inhibited CBL1/CIPK23-mediated phosphorylation of SLAH3. Therefore, PBL27-regulated SLAH3 activity was possibly independent of ABA signaling [87]. It was reported that flg22–FLS2 signaling can activate OST1 which then phosphorylates S-type anion channel SLAC1 to regulate stomatal immunity [45]. However, there is no evidence to show that BIK1, the RLCK downstream of the FLS2 signaling pathway, directly interacts with an anion channel to regulate stomatal immunity (Figure 3).

One of the main functions of apoplastic ROS produced by Rbohs is to regulate stomatal movement [88]. Chitin-elicited plant immune responses also include ROS production [74]. As mentioned above, PBL27 activates a MAPK cascade, but not ROS production in chitin-mediated immunity [84]. However, a study revealed that chitin-triggered ROS production also depends on the phosphorylation of RbohD by BIK1, similar to that of flg22-triggered ROS production in FLS2-mediated immune signaling [53,60]. Intriguingly, OsRLCK185 was involved in both chitin-triggered MAPK activation and ROS production in rice [81]. Although the mechanism by which OsRLCK185 regulates ROS production is still unknown, it is still reasonable to propose that OsRLCK185 may regulate the phosphorylation of Rboh to produce ROS in a way similar to BIK1. It is worth pointing out that OsRLCK185 is required in rice for both ROS production and MAPK signaling triggered by chitin, two immune responses in Arabidopsis mediated by BIK1 and PBL27, respectively, which suggests that the functions of these RLCK VII members are differentiated in chitin-induced stomatal closure.

## 4. LecRKs Mediate Various Signals to Regulate Stomatal Immunity

Lectin receptor kinases are a group of RLKs which contain an extracellular lectin motif predicted to bind various carbohydrates such as oligosaccharides [89]. Plant lectin receptor kinases were classified into three subgroups according to their extracellular lectin motifs: G-type, C-type, and L-type [90]. Because the extracellular domain is similar to soluble legume lectins that are universal in leguminous seeds, L-type lectin receptor kinases (LecRKs) are also known as legume-like lectin receptor kinases [90]. Accordingly, LecRKs are supposed to perceive oligosaccharides released from pectin [89]. A total of 45 LecRKs are encoded by the Arabidopsis genome, which were divided into 9 sub-groups (I to IX) and 7 singletons (LecRK S.1 to S.7) [90]. In the past decade, the functions of some LecRKs have been discovered. Many *LecRK* genes are rarely transcribed during plant development, but their expression levels could be induced by elicitors or pathogens [90], implying that LecRKs may be critical for plant immunity or stress responses.

ATP is generally considered the universal energy currency in organisms. However, ATP released into the extracellular matrix (eATP) functions as a signal molecule to mediate various stress responses and immune responses [91,92]. eATP plays a role in stomatal opening by triggering ROS production in guard cells of the wild type [93]. In contrast, the ATP-induced ROS increase was not observed in *atrbohD/F*. Moreover, the ATP-triggered Ca^2+^ influx and H^+^ efflux in guard cells were significantly suppressed in *atrbohD/F*. Similarly, the Ca^2+^ influx and H^+^ efflux induced by ATP treatment were not detected in *gpa1* mutants [93]. These data demonstrated that eATP functions upstream of ROS production and G-protein to regulate stomatal movement. Similar mechanisms were found in other species. For instance, eATP stimulates stomatal opening in *Vicia faba* by activating Ca^2+^ channels in guard cells [94]. DOES NOT RESPOND TO NUCLEOTIDES 1 (DORN1/LecRK I.9) was the first eATP receptor identified in plants through a forward genetic screen for mutants insensitive to ATP [37]. A recent study indicated that DORN1/LecRK I.9 directly phosphorylated RbohD to mediate the production of ROS in guard cells and induce stomatal closure [95]. Interestingly, the conclusion that eATP signal promotes stomatal closure in this study [95] seems opposite to the previous results [93,94]. It should be noted that the concentrations of ATP used in the physiological experiments were significantly different in these independent studies, implying that the concentration of eATP may be a critical factor in regulating stomatal movement, or that eATP regulates stomata aperture in immunity through various signaling pathways. Microarray analysis revealed that a number of eATP-induced genes that play roles in plant defense responses were also involved in the JA signaling pathway. eATP induced the degradation of JASMONATE ZIM-DOMAIN PROTEIN 1 (JAZ1), a repressor of JA signaling, through the SCF^COI1^-proteasome pathway [96]. Consistent with the previous studies [93,97], the second messengers, such as Ca^2+^, ROS, and NO, were necessary for eATP-activated JA signaling [96]. Based on these results, the eATP–DORN1/LecRK I.9 signaling pathway may mediate plant immunity and stomatal movement by employing JA signaling components.

**Figure 4:**
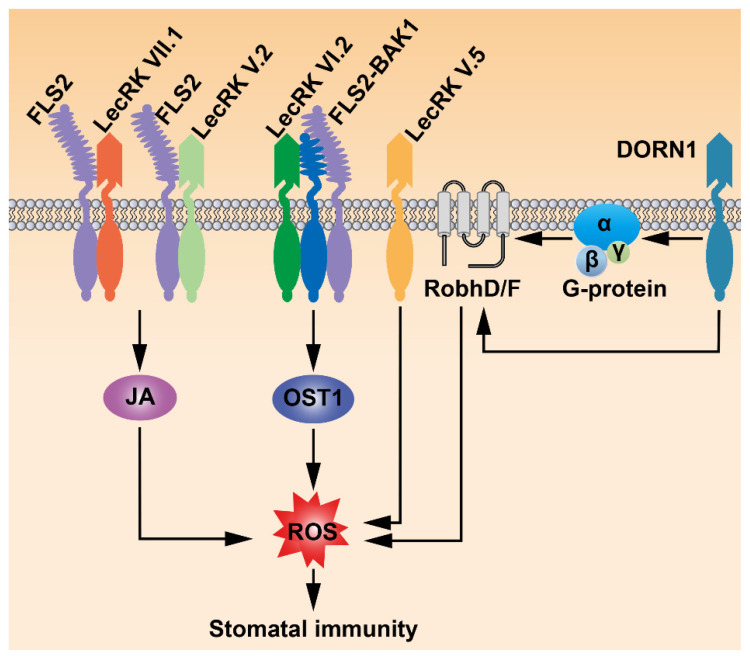
LecRKs regulate stomatal immunity. DOES NOT RESPOND TO NUCLEOTIDES 1 (DORN1/LecRK I.9) perceives extracellular ATP (eATP) to mediate the production of ROS in stomatal closure through G protein-dependent or -independent pathways. LecRK-VI.2 associates with the FLS2 PRR complex to regulate PAMP-induced stomatal immunity. LecRK-V.5 regulates stomatal immunity upstream of ABA-mediated ROS burst. LecRK-V.2 and LecRK-VII.1 function in a nonredundant way to regulate stomatal movement in immunity through the JA signal pathway.

Nicotinamide adenine dinucleotide (NAD^+^) was considered the precursor of the second messenger cyclic ADP-ribose (cADPR) that triggers Ca^2+^ signaling in organisms [98,99,100]. Despite the fact that NAD^+^ functions as a signal molecule, the potential receptor of NAD^+^ in plants was not found until Arabidopsis LecRK-I.8, a homolog of DORN1/LecRK-I.9, was identified to sense extracellular NAD^+^ (eNAD^+^) [101]. Exogenous NAD^+^ application induced *PR* gene expression in the wild type but failed in the *lecrk-I.8* mutants [101,102]. The *lecrk-I.8* mutants exhibited significantly elevated susceptibility to a low concentration of bacterial pathogen *P. syringae* pv. *maculicola* (*Psm* ES4326) [101]. Furthermore, LecRK-I.8 specifically bound with NAD^+^. Therefore, LecRK-I.8 functions as a potential receptor of eNAD^+^ to play a positive role in plant immunity. LecRK-VI.2 is the second potential receptor of eNAD^+^ as well as extracellular NAD^+^ phosphate (eNADP^+^), which plays a key role in the biological induction of systemic acquired resistance (SAR). LecRK-VI.2 interacted with BAK1 both in vivo and in vitro [103], and associated with FLS2 upon flg22 elicitation [36], implying that LecRK-VI.2 may be required for plant PTI. Several PTI responses including stomatal closure triggered by flg22 were impaired in the *lecrk-VI.2-1* mutants [104]. Based on these results, a NADP^+^ signal is necessary for flg22–FLS2/BAK1-mediated stomatal immunity although the detailed mechanisms need to be further explored in the future (Figure 4).

Loss of *LecRK-V.5* function increased plant resistance to *Pst* DC3000. Fewer bacteria and disease symptoms were observed in the *lecrk-V.5* mutants than the wild type because the stomata were closed in the mutants during the bacterial invasion, suggesting that LecRK-V.5 negatively regulates stomatal immunity [105]. Consistently, *LecRK-V.5* is expressed in stomatal guard cells. *Pst* DC3000 treatment failed to re-open the stomata of the *lecrk-v.5* mutants while transgenic lines overexpressing *LecRK-V.5* did not show PAMP-triggered stomatal closure [105]. Moreover, significantly elevated levels of ROS in guard cells were detected in the *lecrk-v.5* mutants, whereas overexpression of *LecRK-V.5* resulted in reduced ROS production after ABA treatment [105]. Collectively, LecRK-V.5 negatively regulates stomatal immunity and functions upstream of ABA-mediated ROS burst (Figure 4). Two other LecRKs, LecRK-V.2 and LecRK-VII.1, are also expressed in stomatal guard cells. The *lecrk-v.2* and *lecrk-vii.1* mutants showed more susceptibility to *Pst* DC3000 because stomatal closure of these mutants was defective when inoculated with *Pst* DC3000. On the other hand, the *lecrk-v.2*/*vii.1* double mutants showed similarly increased susceptibility when compared with both single mutants [106]. Taken together, LecRK-V.2 and LecRK-VII.1 control stomatal immunity independent of each other. Both LecRK-V.2 and LecRK-VII.1 were part of the FLS2 PRR complex, and the interactions between LecRK-V.2/VII.1 and FLS2 could be induced by flg22 [106]. Although the guard cells of both *lecrk-v.2* and *lecrk-vii.1* mutants exhibited similar responses to ABA as the wild type, MeJA failed to induce stomatal closure in these mutants. In addition, ROS production in guard cells triggered by flg22 was not observed in the *lecrk-V.2* and *lecrk-VII.1* mutants. These results indicated that LecRK-V.2 and LecRK-VII.1 may function together with FLS2 to control stomatal immunity in a nonredundant manner through the JA signaling pathway (Figure 4).

Arabidopsis LecRKs are involved in a variety of biological processes, including development and responses to stimuli. For example, LecRK-IV.2 was identified to regulate pollen development [107]. LecRK-VIII.2 functions upstream of a MAPK cascade to control seed size and number [108]. More and more LecRKs were found to play crucial roles in plant immunity [36,105,106,108,109]. Interestingly, although these LecRKs share similar extracellular lectin motifs, they regulate immune responses in distinct pathways, suggesting that the functions of these LecRK members differentiated during the process of evolution. For example, LecRK-V.5 negatively regulates stomatal closure in immune response [105], whereas LecRK-V.2 plays a positive role in FLS2-mediated stomatal immunity [106]. Intriguingly, their homologous LecRK-V.7 is involved in LPS-mediated immunity [110]. On the other hand, the functional diversification of LecRKs is also embodied in various signal molecules that they sense in response to pathogen invasion. For instance, DORN1/LecRK-I.9 perceives the eATP signal; LecRK-I.8 senses eNAD^+^; LecRK-VI.2 is involved in the perception of eNAD^+^ and eNADP^+^ [37,101,103]. It is worth noting that both LecRK-I.8 and LecRK-VI.2 can sense the eNAD^+^ signal. How the specificity of these two LecRK-mediated eNAD^+^ signaling is determined needs to be investigated in the future. It appears that the major roles of LecRKs that have been studied are to regulate plant immunity. What possible immune responses are regulated by the remaining LecRKs, are worth further exploration.

## 5. Malectin-Like Receptor Kinases Function with FLS2 to Mediate Stomatal Immunity

Plant malectin-like receptor kinases possess a characteristic extracellular malectin-like domain that is proposed to recognize oligosacharrides, glycosylated proteins, and cell wall degradation products [111,112]. These RLKs are also known as *Catharanthus roseus* receptor-like kinase 1-like proteins (*Cr*RLK1Ls). They are involved in many biological processes including development, sexual reproduction, plant immunity, and stress response [30,113,114,115,116]. FER was reported to control male-female interactions during pollen tube reception in Arabidopsis [114]. FER also plays an important role in plant immunity. The enhanced accumulation of ROS, flg22-triggered MAPK activation and callose deposition were observed in the *fer* mutants, and the *fer* stomata were constitutively closed, which led to less bacterial proliferation than the wild-type plants [117]. Thus, FER may associate with FLS2 to regulate various plant immune processes, including stomatal immunity.

RAPID ALKALINIZATION FACTOR 1 (RALF1) was identified to function as a peptide hormone perceived by FER to regulate plant cell expansion [118], suggesting that FER may function as a receptor to sense other RALFs in regulating plant immunity. A forward genetic screen identified RALF23 that functions as a negative regulator in plant immunity [119]. RALF23 treatment or overexpression inhibited PAMP-triggered ROS production in plants and increased their susceptibility to *Pst* DC3000 *cor*^–^. However, RALF23 failed to inhibit elf18-induced ROS production in the *fer* mutants, indicating that RALF23 is specifically involved in FLS2-mediated plant immunity. The binding of RALF23 disturbed FER-mediated complex formation between FLS2 and its co-receptor BAK1, which suggested that FER acts as a RALF-regulated scaffold for PRR complexes and thus positively regulates immunity [119]. The phytotoxin COR can hijack the host JA signaling pathway as a structural mimic of JA-Ile to re-open stomata for the invasion of more bacteria into the host plant [17]. A great number of genes with overlapping expression upregulated in both *fer* and COR/JA-induced seedlings were identified, suggesting that FER negatively regulates COR/JA signaling. FER interacted with and phosphorylated MYC2, resulting in the destabilization of MYC2. Furthermore, the phosphorylation of MYC2 was RALF23-dependent [30]. In summary, the RALF23–FER–MYC2 signaling pathway is necessary for COR-mediated host disease susceptibility.

## 6. Conclusions and Future Perspectives

For many pathogens, entry into the apoplastic space is the first critical step to infect the host plant. Stomata are the main entrance for many pathogens to enter into the host tissues. Therefore, immunity occurring in guard cells is the first line of defense against bacteria or fungi. Although the importance of stomata in immune processes has long been recognized [120,121], the mechanism was not revealed until it was found that stomatal closure induced by bacteria requires FLS2 and the guard cell-specific OST1 kinase [15]. This discovery indicated that stomatal movement during bacterial invasion is an important part of plant innate immunity. Plant RLK signaling pathways function in a variety of biological processes, including the defense responses to microbial signals [38,122,123]. Many RLKs, such as PRRs (FLS2, CERK1, EFR), LecRKs, and *Cr*RLK1Ls, were reported to regulate stomatal immunity. As discussed above, the mechanisms of these RLKs regulating stomatal movement in immunity have been investigated in detail.

Recently, several studies deciphered some other RLK-mediated signal pathways involved in the regulation of stomatal movement. GUARD CELL HYDROGEN PEROXIDE-RESISTANT 1 (GHR1) is a member of the Arabidopsis LRR-RLK subfamily. The stomata of *ghr1* mutants showed impaired responses to elevated CO_2_, ABA, light-dark transitions, and flg22. GHR1 activated SLAC1 via forming a complex with CALCIUM-DEPENDENT PROTEIN KINASE 3 (CDPK3) and SLAC1 [124,125]. Although current data showed that GHR1 is necessary for flg22-triggered stomatal immunity [125], whether GHR1 functions downstream of FLS2 remains to be clarified. Arabidopsis KINASE 7 (KIN7) was assumed to be a receptor of LPS [126], that phosphorylates TANDEM PORE K^+^ CHANNEL 1 (TPK1) to regulate ABA- and CO_2_-mediated stomatal closure [127]. RECEPTOR-LIKE PROTEIN KINASE 1 (RPK1) was supposed to be a positive regulator in ABA signaling. Lack of *RPK1* impaired ABA-induced stomatal closure, while overexpression of *OST1* completely rescued the defects of *rpk1* in response to ABA [128]. Arabidopsis STRESS INDUCED FACTOR 2 (SIF2) directly interacted with the FLS2-BAK1 PRR complex and SLAC1 and phosphorylated SLAC1 to mediate ABA-mediated stomatal immunity [129]. Although the detailed mechanisms mediated by these RLKs still need to be investigated, it looks like many of them function in stomatal immunity through regulating the activities of various ion channels directly or indirectly. The fact that so many RLKs were identified to regulate stomatal immunity also reminds us that plants are facing numerous environmental stimuli and there must be more RLKs involved in stomatal immunity. These RLKs responding to different environmental stimuli need to be identified, and their detailed mechanisms regulating stomatal immunity should be investigated in the future.

It has been revealed that various extracellular signals contribute to stomatal immunity. These signals include PAMPs, extracellular purines, and peptide hormones, which are usually sensed by RLKs. For instance, FLS2 and DORN1/LecRK-I.9 directly perceive the flg22 and eATP signals, respectively, to regulate stomatal immunity [15,37,42,43,95]. Possibly, distinct RLK receptors are employed to sense various PAMPs from different pathogens. At the same time, pathogen invasions lead to damages of plant cells, generating various infection-related molecules such as peptides and oligosaccharides that need to be sensed by a variety of RLK receptors to coordinately control stomatal responses. Multiple RLK-mediated signaling pathways make it reliably effective to guard pathogen invasion during stomatal immunity. It should be pointed out that the ligands of many RLKs in stomatal immunity, such as GHR1, LecRK-V.2, LecRK-VII.1, have not been identified yet. Some studies reported that several other RLKs regulating stomatal movement were associated with the FLS2 PRR complex, such as LecRK-V.2, LecRK-VII.1, FER, and SIF2 [106,119,129], which suggested that FLS2 may play a central role in stomatal immunity by integrating these signaling pathways. On the other hand, these RLKs and FLS2 may form a specific receptor complex to mediate the flg22 signal, which is at least partially supported by the results that flg22-triggered immune responses in guard cells were not detected in *lecrk-V.2* and *lecrk-VII.1* [106].

Usually, the FLS2-mediated signaling regulates stomatal immunity by ultimately modulating the activities of transporters or channel proteins localized in the plasma membrane through multi-pathways downstream of FLS2 to control stomatal aperture during the immune response [45,53,66,73]. One of these pathways is the MAPK signaling cascade. MAPK cascades were reported as key components downstream of FLS2 to regulate plant immunity. The MEKK1–MKK4/5–MPK3/6 cascade is downstream of FLS2 to modulate Arabidopsis resistance to pathogens [130]. The MEKK1–MKK1/2–MPK4 cascade downstream of FLS2 contributes to plant PTI [131,132]. Similarly, MAPK signaling also plays a critical role downstream of FLS2 in stomatal immunity. For instance, decreased expression of *MPK3* resulted in stomatal movement insensitive to ABA and H_2_O_2_ [133]. Recent studies demonstrated that MKK4/5 and MPK3/6 transduce the flg22 signal and play crucial roles to control stomatal immunity by regulating the metabolism of osmolytes [86] and modulating actin remodeling via phosphorylation of VILLIN3 (VLN3) [134]. Some lines of evidence indicated that RLKs can phosphorylate transporters or channels directly or activate these membrane-localized protein machineries by RLCKs which are associated with RLKs. For example, CANALIZATION-RELATED, AUXIN-REGULATED MALECTIN-TYPE RLK (CAMEL), and CANALIZATION-RELATED RECEPTOR-LIKE KINASE (CANAR) directly activate PINFORMED (PIN) proteins that are auxin transporters to coordinately mediate auxin polarization [135]. Cyclic nucleotide-gated channel proteins CYCLIC NUCLEOTIDE GATED CHANNEL 2 (CNGC2) and CNGC4 are phosphorylated by BIK1 to increase the [Ca^2+^]_cyt_ during pathogen invasion [136]. This non-genomic function paradigm also exists in RLK-mediated stomatal immunity possibly because stomatal movement triggered by pathogen invasion is a rapid response. For instance, FLS2 phosphorylates channel proteins directly or through RLCK and downstream kinases such as OST1 [48,73] (Figure 2). Therefore, other RLKs without known mechanisms, especially those LecRKs involved in stomatal movement, may regulate stomatal immunity through phosphorylating the targeting channel proteins or ion transporters after they perceive the signals during pathogen invasion. However, the downstream signaling components of these RLKs, such as co-receptors, RLCKs, MAPK cascades, and the possible targeting ion channels or transporters need to be identified.

Environmental factors, such as humidity, light, and temperature, also affect stomatal movement, and they often share similar downstream signaling events with PRR-triggered stomatal immunity, including phytohormone signaling, ROS production, and activation of channel proteins [71,72,137]. Environmental factors inducing stomatal opening may facilitate the invasion of pathogens. For example, air relative humidity (RH) is a major environmental factor affecting stomatal aperture. High RH may act as a suppressor of stomatal immunity, together with anti-stomatal defense factors such as coronatine, to benefit bacterial infection [138]. Whether RLKs involved in stomatal immunity play roles in regulating environmental factor-induced stomatal movement is an intriguing question. GHR1 functions in flg22-triggered stomatal closure [124,125], which is also necessary for stomatal closure caused by low-air-humidity-induced leaf-to-air vapor pressure difference [139], suggesting that environmental stimulus-induced stomatal movement and stomatal immunity may be regulated by the same RLK. The detailed mechanisms of how RLKs function to cope with the abiotic factor-suppressed stomatal immunity, however, need to be explored in the future.
